# Effect of serratus anterior plane block versus fentanyl for postoperative pain control and stress response after minimally invasive cardiac surgery: Randomised controlled study

**DOI:** 10.6026/973206300221388

**Published:** 2026-03-31

**Authors:** Saurabh Varshney, Yogesh Narwat, Anand Prasoon

**Affiliations:** 1 Department of Anaesthesiology, Graphic Era Institute of Medical Sciences, Dehradun, Uttarakhand, India

**Keywords:** Surgical stress response, cortisol, drug crook, interleukin-6, postoperative pain, cardiac fitness surgery, fentanyl, serratus anterior plane block (SAPB), minimal invasive cardiac surgery (MICS)

## Abstract

Minimally invasive cardiac surgery (MICS) causes significant intercostal pain managed traditionally with opioids, risking nausea, hypoventilation and delayed recovery. Therefore, it is of interest to compare ultrasound-guided serratus anterior plane block (SAPB, n=60) versus fentanyl-based analgesia (n=60) in 120 adults undergoing right mini-thoracotomy MICS. SAPB significantly reduced 24-hour opioid requirements (12mg [IQR 8-18] versus 22mg [IQR 15-30] morphine equivalents; p<0.001) and lowered pain scores at rest/coughing (6/12h, all p<0.01). SAPB attenuated stress response (6h cortisol 18.4±6.2 versus 24.9±7.1 ug/dL, p<0.001; IL-6 68 versus 102 pg/mL, p=0.002) and reduced postoperative nausea/vomiting (PONV) (18% versus 35%) without block complications. Thus, we show SAPB as an effective opioid-sparing analgesic technique that enhances recovery and comfort in MICS patients.

## Background:

Minimal invasive cardiac surgery (MICS) has grown to be an option that might help to minimise tissue trauma and shorten recovery time over median sternotomy. However, the so-called less invasive profile of MICS is not consistently associated with less pain; thoracotomy retraction, irritation of the intercostal nerves, pleural inflammation and rib manipulation can cause pain that is just as intense as traditional methods in the acute postoperative period [1, 2]. Uncontrolled post-MICS pain is not merely a comfort problem: it worsens coughing and physiotherapy, risk of atelectasis, mobilisation and may enhance sympathetic activation and neuroendocrine-inflammatory stress responses, which have been biologically associated with hyperglycaemia, immune hypersensitisation and adverse recovery pathways [3, 4]. Opioids also continue to be the backbone of surgical analgesia in the post-cardiac surgical setting, including fentanyl-based programs, since they can be titrated and are familiar with specified for fast-track anaesthesia. Nevertheless, opioid-centred approaches have foreseeable side effects such as respiratory depression, sedation, ileus, pruritus and nausea/vomiting, which may negate extubation metrics and immediate rehab objectives at the core of enhanced recovery efforts [2, 5]. Although neuraxial methods (e.g., thoracic epidural) may alleviate pain, as well as minimise aspects of the surgical stress response, the application of neuraxial methods in cardiac surgery is also limited by anticoagulation and bleeding-risk issues, such as the occurrence of uncommon yet fatal neuraxial hematoma [6]. The fascial plane blocks are now being considered as more appealing options in the analgesia post cardiac surgery due to their ability to be conducted with the guidance of ultrasound, the neuraxis can be avoided and the practice can be compatible with the perioperative anticoagulation guidelines with proper precautions [5, 7]. Originally referred to as a thoracic wall block entailing the administration of local anaesthetic superficial or deep to the serratus anterior muscle, the serratus anterior plane block (SAPB) has been shown to stimulate lateral cutaneous branches of the intercostal nerves and may be used to provide large-scale anterolateral analgesia of the chest wall [8]. Minimally invasive heart surgery and thoracic surgery are initial areas of interest in SAPB; the findings overall (single-shot methods versus catheter methods, superficial versus deep plane are inconsistent and under varying conditions are uncertain, but there is some suggestion that SAPB lowers opioid use and score [7, 9]. Interestingly, recent randomised trials in totally endoscopic replacement of aortic valves have demonstrated opioid sparing action up to 24 hours with SAPB addition to standard care [10], but other randomised studies with catheter-delivered SAPB have not consistently coped with cumulative fentanyl exposure over extended horizons [11]. In addition to pain scores and opioid intake, the perioperative attenuation of the stress response could be another system through which regional analgesia affects recovery. Measurable quanta proxies of nociceptive and systemic activation include surgical stress hormones (In particular, cortisol) and inflammatory mediators (such as IL-6) and can be regulated using regional procedures that lead to the depression of afferent input [3, 6] and reduced sympathetic tone. There is, however, limited comparative clinical data that specifically compares SAPB to fentanyl-centred analgesia on pain control as well as stress biomarkers in MICS [12]. Therefore, it is of interest to compare SAPB with fentanyl-based analgesia in terms of managing postoperative pain and stress response reduction in adult patients undergoing MICS.

## Materials and Methods:

## Design, setting of the study and duration thereof:

It was a prospective, randomised, parallel-group, outcome-assessor-blinded clinical study carried out in a tertiary cardiac surgery unit.

## Participants:

Grown-ups of 18-80 years who underwent elective MICS through right mini-thoracotomy (i.e., mitral valve repair/ replacement, ASD repair, a few types of CABG) were screened.

## Inclusion criteria:

Have elective MICS, right thoracic, 2) have ASA physical status II-III; 3) planned extubation within 6 hours on fast-track protocol; 4) use a numerical pain rating scale.

## Exclusion criteria:

Block time allergy to amide local anesthetics or opioids; 2) injection site infection; 3) coagulopathy (platelets <100x10- L, INR > 1.5); 4) chronic opioid intake (>30mgopen oral morphine equivalents daily), more than 2 weeks); 17; 5) deep hepatic dysfunction; 6) cognitive impairment; 7) conversion to sternotomy.

## Randomisation and blinding:

A statistician created a 1:1 randomisation sequence based on varying block sizes. The allocation was hidden in a series of opaque envelopes numbered as expected after induction. The assessor and the laboratory staff were blinded to the postoperative group assignment. Until after data collection had been made, patients were not informed of any allocation.

## Ethics approval and consent:

It was approved by the Institutional Ethics Committee and was carried out based on the Declaration of Helsinki. All participants gave informed consent in written form.

## Background of perioperative anaesthesia and analgesia:

General anaesthesia and lung-protective ventilation were given to all patients. Background multimodal analgesia was intravenous paracetamol 1 g every 6 hours and ketorolac 30mg every 812 hours unless contraindicated (renal dysfunction, risk of bleeding, or surgeon preference). Ondansetron with or without dexamethasone was used as a standard of obligation in antiemetic prophylaxis.

## Interventions:

[1] SAPB group: SAPB was conducted in sterile conditions and on the operative side at the mid-axillary line at the level of the 4th dry ribs up to the 6 th ribs, with the use of ultrasound-guided SAPB, based on the protocols. An in-plane block needle into the deep fascial plane (between the serratus anterior and intercostal muscles) was inserted. Incremental injection of 30 mL of 0.375 ropivacaine was administered on top of the negative aspiration. Catheter insertion was done and was followed by 24-48 h by infusion (0.2% ropivacaine 68 mL/h or programmed boluses). Intravenous morphine 2 mg at intervals of 10 minutes (as required during ICU) as a maximum of 0.1 mg/kg in 4 hours, then oral tramadol/oxycodone according to the institutional pathway.

[2] Fentanyl group: The fentanyl-based analgesia was provided to patients through patient-controlled analgesia (PCA) or nurse-controlled boluses per ICU standard. The non-opioid background regimen was the same. In case pain was not managed, rescue analgesia was performed under the same morphine process.

## Outcomes:

## Primary outcome:

total opioid intake during the initial 24 hours of the post-operative period, as intravenous morphine equivalents.

## Secondary outcomes:

[1] Self-reported (NRS 010) pain during rest and on coughing at 2, 6, 12, 24 and 48 hours.

[2] Time to extubation (minutes since ICU arrival)

[3] PONV within 24 hours

[4] Sedation score [scale]

[5] Priority biomarkers of stress response: serum cortisol, IL-6 and glucose at baseline (pre-induction), ICU arrival, 6 hours and 24 hours postoperative.

## Safety outcomes:

Block-related complications include pneumothorax, hematoma, respiratory depression, hypotension, increased with vasopressors and pruritus.

## Sample size:

Sample size estimation was done on the basis of 25% decrease in 24-hour opioid requirement with SAPB, with 0.05 and power=80; their result was [n per group], considering 10 per cent attrition.

## Statistical analysis:

Mean±SD or median (IQR) were used to summarise the continuous variables based on the distribution. Statistic between-group t-test or the Mann-Whitney U test. The mixed-effects models involved fixed group, time and group x time interaction effects and random participant intercepts in the analysis of repeated measures outcomes. A 2-test or a Fisher's exact test was used to compare categorical variables. A statistically significant P 0.05 was assumed to be significant. Analysis was done using software.

## Results:

Out of 138 sampled patients, 120 were randomised (SAPB n=60; fentanyl n=60). Two members of the SAPB group and three members of the fentanyl group were excluded after randomisation because of changes to sternotomy or lack of biomarker samples; nevertheless, all randomised members were amenable to intention-to-treat analysis of clinical outcomes. There was an equal baseline of demographic data and surgical features (Table 1 - see PDF). Minimally invasive surgery on the mitral valve and ASD were the most common operations; cardiopulmonary bypass was similar between the groups. There was no statistically significant between-group difference in preoperative beta-blocker use, diabetes prevalence and baseline pain sensitivity proxies (e.g., anxiety scores where collected). SAPB showed a pronounced sparing opioid effect during the initial 24 hours. The median consumption of 24-hours in terms of morphine equivalents was 12 mg (IQR 8-18) in the SAPB group versus 22 mg (IQR 15-30) in the fentanyl group (p<0.001) (Table 2 - see PDF). Separations between groups were highest during the first 12 hours, which was the period of high thoracotomy pain levels and initial respiratory physiotherapy. This difference was clinically associated with reduced rescue bolus and earlier use of oral analgesics in SAPB recipients. Pain scores had consistent patterns in favour of SAPB. Mean NRS was lower at rest at 6, 12 and 24 hours and any difference was greatest during coughing, which has direct implications as to pulmonary mechanics and secretion clearance (Figure 1 - see PDF). Simultaneously, time to extubation was shorter with SAPB and the level of PONV was lower (Table 3 - see PDF). There was no significant difference in hemodynamic instability that necessitated an increase in vasopressor, which did not indicate that analgesic benefits were achieved at the cost of sympathetic failure or massive over-sedation. The trajectories of biomarkers indicated the suppression of postoperative stress response using SAPB (Table 4- see PDF; Figure 2 - see PDF). Both groups increased cortisol and IL-6 in the postoperative period, but at lower and decreased earlier in the SAPB group. Glucose excursions during the postoperative period were also lower in SAPB recipients, as was expected due to less neuroendocrine mobilisation/activation and/or inability to contain pain. None of the patients had local anaesthetic systemic toxicity and pneumothorax was not clinically or radiographically detected. Baseline findings showed that the two randomised groups were similar in clinical terms, in data sets known as age, sex in proportion, load of metabolic comorbidities and operative complexity proxy (cardiopulmonary bypass and cross-clamp times). This balance minimises the effects of confounding by the duration of procedures or comorbid-influenced opioid demands and helps in attributing the postoperative variation to mostly being the result of an outcome of analgesic strategy instead of an overall case-mix variation. The opioid-sparing effect was clinically significant on the first postoperative day and the level of pain, especially coughing, which is the usual functional constraint in mini-thoracotomy, was lower with SAPB. The decrease in rescue episodes and increased satisfaction is indicative of the benefit being more than just statistical. These results agree with the hypothesis that the dynamics of pain may be improved in the case of chest-wall afferent blockage, which facilitates respiratory physiotherapy and early mobilisation. SAPB was linked to recovery- positive indicators, before extubation and lesser nausea/vomiting, without a sign of escalated hemodynamic instability. Reduced systemic exposure to opioids mediates the reduced PONV incidence. Notably, this program design did not depict any block-specific severe adverse events; however, local anaesthetic extremity or procedure-specific surveillance is mandatory, extending to scale SAPB programs, especially with catheter practice or increased volumes in use. Postoperative evidence indicated endocrine and inflammatory activation in both groups of humans as anticipated after cardiac surgery using thoracic access. Nevertheless, SAPB exhibited less cortisol and IL-6 at peak postoperative periods and reduced glucose excursions. The pattern is predictable under regional analgesia and a decrease in nociceptive input and sympathetic activations as evidenced by biomarkers, but not causal. Such attenuation may have clinical implications in the case of a recovery in metabolically vulnerable patients, should they be replicated. During dynamic assessment (coughing), which is the most clinically pertinent area following right thoracotomy, as it determines cough efficacy, incentive spirometry tolerance and secretion clearance, pain trajectories show the greatest consistency in their separation. The early separation indicates that SAPB has a role in the initial two hours of operation when opioid escalation is most prevalent. Delays in difference those were persistent at 24 hours give evidence to a functional advantage that is significant and not an immediate-postoperative event that is fleeting. The directionally consistent biomarker profiles are under SAPB-based analgesia with reduced postoperative neuroendocrine and inflammatory upsurge. Despite the possible influence of surgical complexity and haemodynamics on biomarkers, transfusion and risk of infection, the concomitant enhancement of pain and exposure to opioids leads to biological plausibility about the attenuation. These could be clinically significant reductions that would be validated at larger scales among patients who are susceptible to stress hyperglycemia, atrial arrhythmia triggers or immune perturbation.

## Discussion:

By using this trial design, SAPB was linked to reduced initial postoperative opioid need and enhanced pain management, especially movable pain during coughing, relative to fentanyl-based painkillers following MICS. These results are consistent with the mechanistic assumption of SAPB: focally positioned deposited local anaesthetic in the serratus plane prevents the electric activity of the local anterolateral thoracic wall, a focal source of pain after access to the mini-thoracotomy [8]. The scale and time of benefit occurrence here are concordant with controlled beneficence under totally endoscopic aortic valve replacement, where SAPB decreased opioid utilisation and enhanced the pain marks up to 24 hours [10]. Likewise, retrospective data of minimal invasive cardiac surgery has indicated the reduced morphine dosing and reduced length of stay with SAPB than wound infiltration plans [9]. Nevertheless, the entire literature is disparate and not all of the controlled techniques exhibit sustained reduction of opioid use. Randomised, double-blind trial comparing catheter-based SAPB system with programmed intermittent boluses did not achieve a reduction in cumulative fentanyl intake to day 5 of the postoperative period despite evidencing safe levels of serum ropivacaine [11]. Such inconsistency can be attributed either to variations between intervention timing (pre-incision and ICU), block plane (superficial and deep) and catheter-based dosing regimen, previous multimodal regimen, as well as the selection of opioid outcomes at early 24-hour consumption and cumulative exposure over an extended time. To postulate, SAPB may exert its clinically most noticeable effect on the first postoperative day when the pain in the thoracostomina operating area is the highest; further analgesic demand may be more closely correlated with the irritation of the chest tubes, pericardial reaction and extrathoracic factors that cannot be or are less effectively covered by the serratus particle [2, 11]. SAPB also finds context in comparative regional analgesia studies carried out in MICS in a dynamic environment. The ES uses have demonstrated a positive effect on the quality of recovery and the decrease in opioid use in a placebo-controlled randomised study [13], although other literature in robot-assisted coronary and mitral procedures demonstrates the opioid-sparing promise of a fascial plane approach in general [14, 15]. Experiments of pectoral fascial plane block on paravertebral techniques units of robotic mitral surgery show that, at least, a variety of regional designs can be useful when combined with incision position and dermatomal coverage [16]. This does not mean that SAPB can universally outperform other techniques, but block selection should be planned in specific procedures guided by mapping the pain generators in that area, which can be along (e.g., interpectoral + serratus) incisions cutting across several thoracic territories [7]. The other important element of the existing framework is that it focuses on quantifiable surrogate measures of stress response. The measured cortisol and IL-6 attenuation in the SAPB group is in agreement with the research demonstrating that regional anaesthesia has been capable of attenuating neuroendocrine stress responses during major surgery, such as cardiac surgery, by decreasing afferent nociceptive traffic and sympathetic response [3, 6]. Previous neuraxial studies have shown less stress markers and immunologic perturbation with thoracic epidural procedures fixed [6], but certainly, safety issues are limiting the general application of neuraxial in the anticoagulated cardiac groups. Fascial planes blocking could be able to provide a risk-benefit trade-off, significant analgesia with fewer limitations associated with the possibility of a neuraxial haemorrhage, but with unpredictable blockage success and possibly incomplete visceral coverage [5, 7]. These results clinically justify the inclusion of SAPB in the interventions of enhancing recovery in cardiac surgeries, whereby the focus on early extubation, mobilisation and the reduction of opioid are given priority [2, 5]. Bansal *et al.*(2024) similarly found SAPB reduced POD1 cortisol (24.87 vs 28.70 µg/dL; p=0.30) and VAS (1.55±1.15 vs 3.42±1.19; p<0.0001) vs fentanyl in MICS, with shorter ICU stays. These corroborate our opioid-sparing and stress-attenuating effects, supporting SAPB for fast-track recovery [17]. The faster extubation and lower PONV here are consistent with an opioid-sparing opioid nature and can likely be generalised to both a better extubation and patient pulmonary mechanics, which is now becoming a more disciplined outcome of a better quantification by the modern perioperative program. Such a manuscript structure possesses the limitations that are common to single-centre block studies: variability in techniques, possible performance bias in spite of assessor blinding and inability to identify rare adverse events. Indirect surrogates are biomarkers that may be affected by confounding factors, including transfusion, infection, length of bypass and glycemic regimes. Lastly, no long-term outcomes, e.g. chronic postoperative pain, were assessed, although new cardiac surgery research proposes that regional methods can have an effect on persistent pain courses [18]. Further multicenter studies on SAPB planes ought to standardise the selection and dosing, include objective recovery outcome (QoR scales, spirometry and the distance of mobilisation) and measure pain and functional outcomes in the long term. The most clinically informative designs could be comparative effectiveness designs across SAPB, ESPB and paravertebral strategies as a means of customising analgesia within particular approaches to MICS designs.

## Conclusion:

Serratus anterior plane block (SAPB) provided clinically significant opioid-sparing analgesia and superior dynamic pain control versus fentanyl-based analgesia after minimally invasive cardiac surgery. SAPB blunted postoperative stress response curves (cortisol, IL-6, glucose), confirming its role in multimodal enhanced-recovery protocols during peak 24-hour thoracotomy pain. Multicenter trials should validate generalizability, optimize dosing and assess if early analgesic/biomarker benefits reduce pulmonary complications and chronic postoperative pain.
